# Extragenital Müllerian adenosarcoma with pouch of Douglas location

**DOI:** 10.1186/1471-2407-11-171

**Published:** 2011-05-15

**Authors:** Tito S Patrelli, Enrico M Silini, Salvatore Gizzo, Roberto Berretta, Laura Franchi, Elena Thai, Adolf Lukanovic, Giovanni B Nardelli, Alberto Bacchi Modena

**Affiliations:** 1 Department of Obstetrics, Gynecological and Perinatology Sciences; University of Parma, viale Gramsci 14, 43100 Parma, Italy; 2 Department of Pathological Anatomy, University of Parma, viale Gramsci 14, 43100 Parma, Italy; 3 Department of Maternal and Child Health, University of Ljubljana, Slajmerieva, 3-1000 Ljubljana, Slovenia; 4 Department of Gynecological and Human Reproduction Sciences; University of Padua; via Giustiniani 3, 35128 Padua, Italy

## Abstract

**Background:**

Of all female genital tract tumors, 1-3% are stromal malignancies. In 8-10% of cases, these are represented by Müllerian adenosarcoma an extremely rare tumor characterized by a stromal component of usually low-grade malignancy and by a benign glandular epithelial component. Variant that arises in the pouch of Douglas is scarcely mentioned in the medical literature.

**Case Presentation:**

A 49-year-old para-0 woman, was seen at our OB/GYN-UNIT because she complained vaguely of pelvic pain. She had a mass of undefined nature in the pouch of Douglas. A simple excision of the mass showed low-grade Müllerian adenosarcoma with areas of stromal overgrowth. One and a half year after surgery, at another hospital, a mass was detected in the patient's posterior vaginal fornix and removed surgically. Six months later she came back to our observation with vaginal bleeding and mass in the vaginal fornix. We performed radical surgery. The pathological examination showed recurrent adenosarcoma. Surgical treatment was supplemented by radiation therapy.

**Conclusions:**

The case of Müllerian adenosarcoma reported here is the third known so far in the literature that was located in the pouch of Douglas. To date, only two other such cases have been reported, including one resulting from neoplastic degeneration of an endometriotic cyst.

## Background

Malignant stromal tumors account for 1-3% of all female genital tract tumors; 8-10% of these are Müllerian adenosarcomas. Adenosarcomas are rare tumors scarcely reported in the medical literature that associate benign glandular epithelium with a malignant endometrial stromal component of usually low histological grade. Occasionally, a high-grade malignant stromal component may raise a differential diagnosis issue with leiomyosarcomas and carcinosarcomas [1,2].

Adenosarcomas arise more frequently in the uterus [3], but cases with extrauterine locations in the ovaries [4], cervix [5], vagina [6], and peritoneum [7] have also been reported. Only two cases arising in the pouch of Douglas are known so far [8]. The recurrence rate and the medium- to long-term survival rate depend on the grade and mitotic index of the stromal component [9,10] and on the presence of sarcomatous overgrowth [11]. Tumor location is also an important prognostic factor as extragenital tumors have proved to be more aggressive [8,12].

Of great interest is the association between adenosarcoma and endometriosis, especially in extrauterine forms, because the disease-free survival rate in endometriosis-associated tumors is far better than in unassociated mixed Müllerian tumors [13,14].

The standardization of treatment for extrauterine adenosarcoma is difficult because of the limited clinical experience and the variability of its presenting features.

Here we report the third known case of Müllerian adenosarcoma located in the pouch of Douglas together with a review of the literature.

## Case Presentation

B.V., a 49-year-old para-0 woman, was seen at our Hospital's Operating Unit because she complained vaguely of pelvic pain, without any history of endometriosis. In her past medical history, the patient reported appendectomy and bilateral inguinal hernia surgery. The screening tests (Pap-smear, fecal occult blood test, bilateral mammography and bilateral mammary echotomography) that she underwent periodically were normal. During her hospital stay, routine blood tests, a chest X-ray, and vaginal and cervical smears were performed. The gynecological examination was normal, except for some mild pain on digital exploration at the posterior vaginal fornix. There was no vaginal bleeding. Transvaginal sonography revealed a 10 × 6 cm mass of undefined nature in the pouch of Douglas. Tumor marker tests indicated slightly elevated levels of CA19-9 and CA125. The magnetic resonance imaging (MRI) scan and the computed tomography (CT) scan excluded any distant metastatic spread. Based on the patient's clinical picture, we advised radical surgery. She accepted the surgical removal of the mass, but did not agree to the recommended total hysterectomy, bilateral adnexectomy, and pelvic lymphadenectomy. Therefore a simple excision of the mass was planned. During surgery, intraoperative frozen sections were performed that diagnosed a low-grade mesenchymal tumor to define better after fixation and embedding.

**Figure 1:**
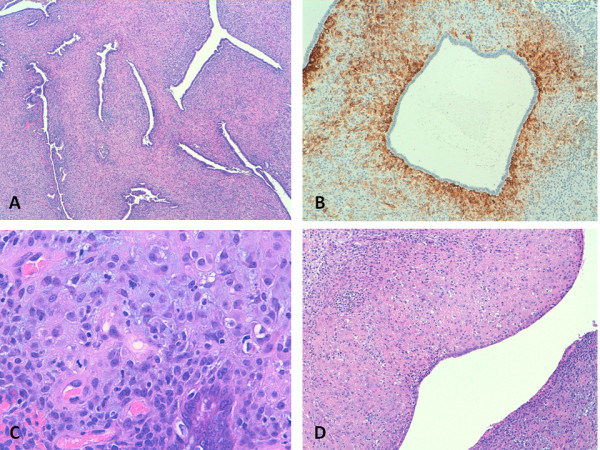
**A. The primary tumor featured a phyllodes-like architecture dominated by an atypical hypercellular stroma forming periglandular cuffs around glandular slit-like spaces lined by endometrial-type cells (4× magnification); B. The sarcomatous component immunostained with CD-10 antibodies (10×) C. and showed scattered mitotic figures (40×); D. Areas of stromal pseudodecidualization were also observed (20×)**.

The final slides showed a biphasic tumor associating epithelial and stromal components. The epithelial component was characterized by endometrial-type cells without atypia showing a variety of metaplastic changes, including ciliated and eosinophilic, that lined polypoid vegetations, and glandular and phyllodes-like structures (Figure 1A). The mesenchymal component was predominantly represented by spindle cells with morphology and immunophenotype similar to proliferative-phase endometrial stroma (vimentin and CD10 positive (Figure 1B); S-100, desmin, H-caldesmon, calretinin, and inhibin negative) and showing mild atypia. cells were arranged in haphazard bundles and in dense periglandular cuffs with intraglandular protrusions. In the latter areas, cells had a mitotic index of 8/10 high-power fields (Figure 1C). Ki-67 proliferation index reached 30% in the most active areas of the sarcomatous component. cells showed high levels of positivity for both estrogen (80%) and progesterone (90%) receptors. Pseudodecidualization of the stroma was also observed in some areas (Figure 1D). Over 30% of the lesion consisted exclusively of stromal proliferation devoid of any epithelial component. These findings were consistent with a diagnosis of low-grade Müllerian adenosarcoma with areas of stromal overgrowth.

The post-operative course was uneventful and after a few days the patient was discharged from hospital.

One and a half year after surgery, a mass was detected in the patient's posterior vaginal fornix. It was removed surgically at another hospital unit and submitted for histology, which confirmed a recurrence of low-grade Müllerian adenosarcoma showing features similar to those of the primary lesion. No significant changes in the proliferative activity of the tumor were observed as assessed by Ki-67 staining. The patient chooses not to receive any adjuvant therapy. Six months later she came back to our observation with vaginal bleeding and a 4 cm polypoid mass in the posterior vaginal fornix. The MRI scan and the CT scan excluded any metastatic disease. Piver's type-III radical hysterectomy with bilateral adnexectomy, selective pelvic lymphadenectomy, and upper colpectomy were performed. Postoperative course regular, without fever. The pathological examination showed recurrent adenosarcoma infiltrating the uterus and the posterior wall of the cervix (tumour dimensions: 4,5 × 3,2 × 2,9 cm; weight: 79 g). The parametria were not involved. The recurrent tumor showed morphological and antigenic features similar to the primary lesion. The lymph node examination was negative for metastasis. There was no evidence of endometriotic foci. Surgical treatment was supplemented by radiation therapy in the pelvic cavity with a total dose of 50 Gy.

## Conclusions

Müllerian adenosarcoma is an uncommon mixed epithelial-mesenchymal tumor of low-grade malignancy that affects the female genital tract. In most cases, it occurs in women aged 14 to 89 years (mean age, 58), is located in the uterus and is accompanied by non-specific symptoms, i.e. vaginal bleeding, uterine enlargement, and vague pelvic pain [15].

Since the first literature report by Clement and Scully [1] in 1974, in spite of the low incidence of this tumor investigators have been able to define its broad range of features and their clinical and prognostic implications.

First of all, it is essential to provide a histological diagnosis of the lesion. Adenosarcoma should be distinguished from its uncommon benign counterpart, adenofibroma, but the differential diagnosis cannot be established with certainty from curettage or biopsy specimens [8].

Histological criteria for malignancy include at least mild atypia with a mitotic count of 2 per 10 HPF, distinctive periglandular cuffs of cellular stroma with or without intraglandular protrusions of stromal element, and invasion [1,11].

Heterologous differentiation is also found in 10-15% of cases and helps in the differential diagnosis. Indeed, a recent paper challenges the existence of adenofibroma, as aggressive tumor behavior has also been reported in cases without sarcomatous overgrowth and with only mild nuclear atypia and a negligible mitotic rate [3].

Once the neoplastic transformation has been established, the major factor affecting the outcome is tumor location. In patients with genital tumors, disease recurrence occurs in about 25% of cases, usually in vagina or in pelvis, with a mortality rate of about 10%. By contrast, extragenital tumors are reported to recur in over 50% of cases and have a mortality rate of about 35% [12].

Extragenital tumors are distinctively less common and are primarily located in the pelvic peritoneum, retroperitoneum, broad and round ligaments, vesicouterine pouch, and rectouterine pouch. Only two cases of Müllerian adenosarcoma located in the pouch of Douglas, described by Ostor et al. [8] have been reported so far (Table 1).

**Table 1:** Summary of cases of extragenital Müllerian adenosarcoma until 2003 and our case, modified from Murugasu[21]

Authors	Age	Location	Treatment	Follow-up
Douglas et al.	18	Retroperitoneum	Chemotherapy	Died after 10 wks with distant metastases

Bard et al.	46	Pelvic peritoneum	Surgery, chemotherapy	Died after 11 wks with distant metastases

Clement et al.	45	Pelvic peritoneum	Surgery, radiation therapy	Pelvic recurrences; died after 9 mths

Clement et al.	73	Pelvic peritoneum	Surgery	Died after 2 mths

Clement et al.	58	Pelvic peritoneum	Surgery	Local recurrence at 15 mths; lung metastases at 45 mths

Kao & Norris	42	Round ligament	Partial surgery, chemotherapy, radiation therapy	Died of disease after 10 mths

Russell et al.	29	Broad ligament	Surgery, radiation therapy	Local recurrence at 5 mths; symptom free; died of melanoma after 9 yrs

Vara et al.	62	Bladder	Surgery	Disease free at 1 yr

Roman et al.	63	Retroperitoneum	Surgery, chemotherapy, radiation therapy	Multiple recurrences; heart and liver metastases; died of disease after 10 yrs

Kerner et al.	32	Broad ligament	Surgery	Pelvic recurrences at 22 mths

De Jonge et al.	16	Pelvic peritoneum	Surgery, chemotherapy	Disease free at 57 mths

Kato et al.	20	Peritoneum	Surgery	Disease free at 1 yr

Visvalinga et al.	50	Pelvic peritoneum	Surgery, chemotherapy	Died of disease after1 yr

Ostor et al.	49	Pouch of Douglas	Surgery, chemotherapy, radiation therapy	Alive with disease at 18 mths

Morugasu et al.	23	Pouch of Douglas	Surgery, chemotherapy, radiation therapy	Disease free at 1 yr

Present case	49	Pouch of Douglas	- First step: partial surgery; - Second step (after 24 mths): radical surgery and radiation therapy	Local multiple recurrences (after 18 and 24 mths), today disease free

Clements and Scully [15] in 1990, in a large case series of 100 uterine adenosarcomas, showed that recurrence was strongly correlated to the degree of myometrial invasion and that it primarily occurred in vagina, pelvis and peritoneum, whereas hematogenous spread was found only in two cases. In the Gynecologic Oncology Group (GOG) study [10], 30% of women had a recurrence of the disease and 20% died over a mean follow-up period of 38.3 months.

In this study, too, we were able to confirm a significant correlation between extrauterine location or myometrial invasion and recurrence rates. On the other hand, the degree of myometrial invasion and the recurrence rate are strongly correlated to the differentiation grade and the mitotic index of the sarcomatous stromal component [9,11].

Sarcomatous overgrowth - as defined by a confluent growth of the sarcomatous component occupying at least one quarter of the tumor - and a deep muscular invasion are considered adverse prognostic factors [9,13].

However, sarcomatous overgrowth is seen more frequently in ovarian than in uterine tumors.

Ultimately, the most important negative prognostic factors are lesion location, the degree of myometrial invasion, and sarcomatous overgrowth. A further strongly unfavorable prognostic factor is disease recurrence following primary treatment [11].

By contrast, a favorable prognostic factor in adenosarcoma patients is the presence of endometriosis. The possibility of a neoplastic transformation of endometriotic foci has been suggested by several studies. In their study, Stern et al. [14] (2001) investigated risk of neoplastic transformation in 1000 patients with endometriosis. they observed that - as expected - endometrioid adenocarcinoma was the most common histotype among degenerative endometriotic lesions, followed - quite unexpectedly - by adenosarcoma, with either ovarian or extragenital location. However, most noteworthy is the fact that in the literature there are reports about cases with sarcomatous degeneration of long-standing endometriotic lesions [4], which are associated with absolute and disease-free survival rates that are considerably higher than those for tumors unassociated with endometriosis. This may be a consequence of lower aggressiveness and a lower mitotic rate of the stromal component, which would explain the lower invasiveness found in these cases [12].

On immunohistochemical testing of endometriosis-associated adenosarcoma, it is frequent to find positivity for estrogen and progesterone hormone receptors. Soslow et al. [16] (2008) also found a correlation with tamoxifen hormone therapy in some forms that expressed progesterone and estrogen receptors, suggesting that estrogen stimulation is somehow involved in tumor pathogenesis, and providing interesting biological clues to the possible efficacy of hormone therapy. Hines et al. [17] (2002) reported a case of extragenital adenosarcoma arising in an endometriotic lesion treated by surgical resection and adjuvant therapy with medroxyprogesterone acetate. The 10-month follow-up of this case did not show either residual disease or recurrence, suggesting that medroxyprogesterone acetate may be proposed as a useful drug in adjuvant hormone therapy for advanced-stage tumors.

Notwithstanding the great progress made in the diagnosis and treatment of Müllerian adenosarcoma, its rarity and its heterogeneous behavior make difficult to set up well-defined therapeutic protocols. Given the risk of recurrence, metastasis and progression to high-grade sarcomas, even low-grade tumors should be carefully considered especially when associated with unfavorable risk factors.

As Müllerian adenosarcoma can evolve into the most aggressive form of all mixed Müllerian tumors [18], i.e. adenosarcomas with sarcomatous overgrowth and poorly differentiated sarcoma not otherwise specified, small-size low-grade malignant lesions with scarce myometrial involvement should never be underestimated, in order not to risk "undertreatment" with all its implications.

Radical surgery remains a therapeutic cornerstone. Shi et al. [2] (2008) recommended a surgical approach similar to that used for the corresponding disease stages of endometrial carcinoma. Total abdominal hysterectomy with bilateral adnexectomy appears adequate for stage-I patients. The decision of giving post-surgery chemotherapy may be based on the extent of muscular invasion and sarcomatous growth. While optimum treatment has yet to be defined for stage-II patients, Guidozzi et al.[19](2000) demonstrated that 34- to 56-month disease-free survival can be achieved in stage-III patients treated by neoadjuvant radiation therapy followed by radical surgery and then again by adjuvant radiation therapy followed by three chemotherapy cycles with carboplatinum and farmarubicin. Huang et al. [12] (2009) proposed treatment with ifosfamide and cisplatinum in the extragenital forms with high sarcomatous growth. Del Carmen et al. [20] (2003) suggested that patients should be treated with liposomal doxorubicin, which is well tolerated and effective, both in post-surgical recurrences and as adjuvant therapy in extragenital forms. Notwithstanding the success obtained in reducing the tumor mass, however, in the case reported by these authors the tumor's highly aggressive behavior led to a rapid resumption of growth at treatment discontinuation and the patient eventually died after 29 months from diagnosis and 20 months from treatment.

Thus, while awaiting to acquire a wider clinical experience on this rare form of genital tumor, we recommend a "customized" treatment that uses surgery and either neoadjuvant or adjuvant chemo/radiation therapy, which, in the event of early diagnosis, can be expected to achieve a disease-free result.

## Competing interests

The authors declare that they have no competing interests.

## Authors' contributions

TSP and SG have made substantial contribution to conception and design. LF and RB have been involved in drafting the manuscript. EMS and ET performed the pathological examination. AL revising it critically for important intellectual content. GBN and ABM have given final approval of the version to be published. All authors read and approved the final manuscript.

## Consent

Written informed consent was obtained from the patient for publication of this case report. A copy of the written consent is available for review by the Editor-in-Chief of this journal.

## Pre-publication history

The pre-publication history for this paper can be accessed here:

http://www.biomedcentral.com/1471-2407/11/171/prepub
